# The potential preventive effect of dietary phytochemicals In Vivo

**DOI:** 10.1038/s41405-023-00157-5

**Published:** 2023-07-18

**Authors:** Mai M. Saleh, Zeinab E. Darwish, Manal I. El Nouaem, Nesrin A. Fayed, Ghada M. Mourad, Omneya R. Ramadan

**Affiliations:** 1 Lecturer Oral Pathology, Faculty of Dentistry, Alexandria University, Alexandria, Egypt; 2 Professor of Oral Pathology, Faculty of Dentistry, Alexandria University, Alexandria, Egypt; 3 Center of Excellence for Research in Regenerative Medicine and Applications (CERRMA), Faculty of Medicine, Alexandria University, Alexandria, 21521 Egypt; 4 Department of Histology and Cell Biology, Faculty of Medicine, Alexandria University, Alexandria, 21521 Egypt; 5 Assistant Professor of Oral Pathology, Faculty of Dentistry, Alexandria University, Alexandria, Egypt

**Keywords:** Oral cancer, Oral cancer detection

## Abstract

**Introduction:**

Chemoprevention refers to using specific substances during oncogenesis. Curcumin and catechins are both polyphenol types of phytochemicals present in curcuma longa and green tea. The effect of curcumin is synergistic with epigallocatechin gallate, the most abundant polyphenol in tea.

**Aim:**

To evaluate and compares the chemopreventive effect of both green tea and curcumin (each individually and in combination) through induction of hamster buccal pouch carcinoma.

**Materials and methods:**

Squamous cell carcinoma was chemically induced in fifty Syrian golden hamsters divided into 5 groups (10 each). The first group was used as a normal control group. The second group received the carcinogenic agent only. The other three groups received green tea, curcumin, and a combination of both, respectively. Flow cytometry, immunofluorescence, and immunohistochemical assays were used to evaluate apoptosis, proliferation, and angiogenesis. ANOVA test was used to analyze the results between the study groups.

**Results:**

The cells of the positive control group (B) resulted in 11.57% apoptosis. In the study groups, treatment of the cells with green tea (C), and curcumin (D) and both of them (E) showed increased apoptosis. The fluorescent image in group B showed an increase of the red fluorescence in the nucleus and cytoplasm of the squamous cell carcinoma cells while groups C, D, and E showed a decrease of the red fluorescence in the nuclei of the squamous cell carcinoma cells. The microvessel density was higher in the positive control group as compared to the treated groups.

**Conclusions:**

The combination of green tea and curcumin has a significant chemopreventive effect against oral carcinogenesis.

## Introduction

Cancer is a major cause of mortality and is characterized by uncontrolled cell division of abnormal cells in a small area of the body, where programmed cell death and proliferation are imbalanced [1]. Despite the significant advances and milestones achieved in cancer treatment modalities, both incidence and mortality rates are disappointingly still high and have not decreased in the last 30 years [2], thus urging more research for developing more effective and less toxic treatment approaches [3]. Traditional chemotherapy is effective, but it also has several side effects that may be dose-limiting, meaning that their severity could force cancer treatment to come to a halt [1]. Furthermore, owing to the non-specific nature of chemotherapeutic drugs, not all malignant cells are always destroyed from the body, which might lead to tumor recurrence [4]. Because of the severe limitations of traditional therapy, oral cancer chemoprevention is currently a research focus, and this interest has markedly increased with the improvement in the illustration of the biology of carcinogenesis and identification of possible molecular targets involving this process [5]. Complementary medicinal preparation can be utilized to target specific cancer cells to inhibit tumor growth, metastasis, and progression without causing major adverse effects [6, 7].

Discovery of the naturally occurring plant-based compounds called phytochemicals has facilitated the development of new treatment strategies for patients that are at risk for or have developed head and neck SCC [8]. Phytochemicals are bioactive compounds produced by plants in their primary or secondary metabolism to protect them from external threats, thereby aiding in their development and reproduction. Spices, fruits, vegetables, and other plant species naturally contain dietary phytochemicals [8]. A meta-analysis of several observational studies found that diets high in plant-based foods have a negative correlation with cancer risk in general and can be used in cancer chemoprevention [9, 10]. Plant consumption containing highly potent phytochemicals can be less expensive and convenient than surgery and chemotherapy when compared to conventional cancer treatments [11]. Consequently, phytochemicals are promising adjuvants that help to decrease the incidence of cancer and decrease the financial medical expenses in developing societies [1]. Researchers are interested in the anticancer efficacy of a wide range of plants against various types of carcinomas. A vast number of natural compounds have been investigated for their ability to cause apoptosis in human cancer cells [12]. These substances are high in polyphenols, which have anti-oxidant, anti-migratory, anti-proliferative, and anti-invasive effects on cancer cell properties, and are thought to inhibit carcinogenesis by acting on downstream signaling pathways [13]. While many compounds have been studied, the compounds from a specific category of phytochemicals, phenolics (resveratrol, EGCG, curcumin, quercetin, and honokiol), are emerging as potent and effective inhibitors of oral carcinogenesis. These compounds have been shown to inhibit head and neck SCC growth through different mechanisms [13]. Research has demonstrated that these compounds can regulate cancer cell proliferation by regulating of multiple cell signaling pathways. They can impede cell cycle progression, induce differentiation and apoptosis, inhibit angiogenesis, and inhibit cancer cell invasive and metastatic properties. They can protect normal cells during treatment and reduce the damage caused by chemotherapy and radiotherapy [14].

Green tea has shown scientifically proven useful medical advantages. Polyphenols account for 36% of the dry tea leaf weight, as well as glycosides, leucoanthocyanins, and phenolic acid. Catechins are a group of polyphenols that include (-)-epigallocatechin gallate (EGCG), (-)-epigallocatechin (EGC), (-)-epicatechin gallate (ECG), and (-)-epicatechin (EC) [15]. Green tea has been shown to have antiviral, anti-inflammatory, and antiallergic properties [16]. The first report of EGCG’s cancer-preventive activity was published in 1987 [17], and since then, the importance of EGCG and green tea in cancer prevention and therapy has become widely recognized [1, 18]. Green tea polyphenols have been shown to inhibit various pathways linked to cancer cell growth, survival, and metastasis [1].

Curcumin, a natural polyphenol, is one of the most investigated biomolecules from Mother Nature. Curcuma longa or turmeric, its natural source, has been utilized in Indian Ayurvedic and Siddha medicine, as well as Chinese medicine, for thousands of years [19]. Curcuma longa has been shown to have anti-inflammatory properties in animal models, with curcumin playing a key role. Because of its phenolic composition, curcumin also serves as an antioxidant. This substance affects glutathione and peroxidase activity in the blood, reduces lipid peroxidation, and scavenges reactive oxygen species [12]. Curcumin’s anti-tumorigenic and chemopreventive properties are its most important properties. Curcumin administered orally or topically, was found to dramatically reduce DNA adducts in a study on turmeric and oral cancer [20]. In peripheral cells, curcumin successfully repairs damaged DNA strands. It also deactivates the carcinogens found in tobacco [21]. Furthermore, “combination chemoprevention”, which can not only increase the potential synergistic efficacy of medications but also reduce the toxicity of the individual agents, with a lower dose treatment in a combination regimen, is a significant consideration [22]. Pointing to the evidence that the primary pathways targeted by these drugs have crosstalk [23], chemopreventive agents exert their action through a diversity of mechanisms including induction of apoptosis, cell proliferation suppression, and angiogenesis inhibition [24].

The apoptotic process can be modulated by dietary chemopreventive substances [25]. Angiogenesis (the development of new blood vessels) is a complex vital process involving angiogenic substances generated by cancer cells and immune cells. It has been linked to the progression, aggressiveness, and metastases of various cancers including oral squamous cell carcinoma (OSCC) [26]. Given these multiple lines of evidence, we hypothesized that a combination of these two agents may provide effective chemoprevention through synergistic effects on the apoptotic pathways and other targeted oncogenic (angiogenic and proliferative) pathways. In this study, 7,12-dimethylbenz(a)anthracene (DMBA), induced oral carcinogenesis animal model was used as a system to assess the efficacy of the combination of green tea and curcumin in chemoprevention at the preclinical level.

## Materials and methods

### Animal model

Our controlled comparative experimental study included 50 Syrian golden healthy male hamsters (Mesocricetus auratus), 5 weeks old, weighing 80–129 g, with no anatomical abnormalities. They were obtained from the holding company for biological products and vaccines (VACSERA), Helwan, Cairo, Egypt. Hamsters were housed in show box cages (Technoplast, Italy), one per cage under the same condition on a regular alternating lighting cycle (12:12 light: dark) at the experimental animal unit in the Medical Technology Center of Medical Research Institute, Alexandria University.

They were quarantined for adaptation purposes for 7 days before starting the experiment. Hamsters were housed at a room temperature of (23 ± 1)°C and relative humidity of (50 ± 5)%. Drinking water and conventional feed were provided by the Medical Research Institute guidelines for the care and use of experimental animals. The Alexandria University review committee approved the animal study and the procedures followed following institutional guidelines (IRB#00010556-IORG0008839).

To establish an oral cancer model, we chemically induced OSCC by painting the left buccal pouch, only to make the opposite side easier to feed on, of the hamsters with 7, 12 dimethylbenz [a] anthracene carcinogen (DMBA, Sigma Aldrich, 57,976 Germany). We used the hamster buccal pouch as our oral cancer model due to the similarities between its lining mucosa and the epithelium covering the hard palate, tongue, and gingiva of the human oral cavity. Furthermore, multiple correspondences to human OSCC were found regarding morphology, molecular markers expression, and finally DNA mutations [27].

All hamsters that fulfilled the inclusion criteria (50 hamsters) were randomly assigned into two groups; control arm A (*n* = 10) and DMBA cancer induction arm (second group) (*N* = 40). The first group (A) was used as a normal control group from which normal buccal pouches were dissected.

The second group received the carcinogenic agent DMBA prepared by dissolving 0.5 g DMBA in 100 ml paraffin oil. Hamsters in the cancer induction group were divided into 4 subgroups (*n* = 10 each): (B) DMBA induction only (considered as the positive control group), (C) receiving green tea (obtained from classic Darjeeling Bud White Teas Pvt, was prepared as solids which were made fresh (6 mg tea solids/ml) and were given to the hamsters as the sole source of drinking), (D) receiving curcumin powder 10 mmol (Alpha Chemika (Mumbai, India), dissolved in 100 ml paraffin oil and applied topically on the left buccal pouch three times/week in alternative days with the carcinogen), and (E) combination of both in same concentrations mentioned.

### Treatment plan

**Fig. 1:**
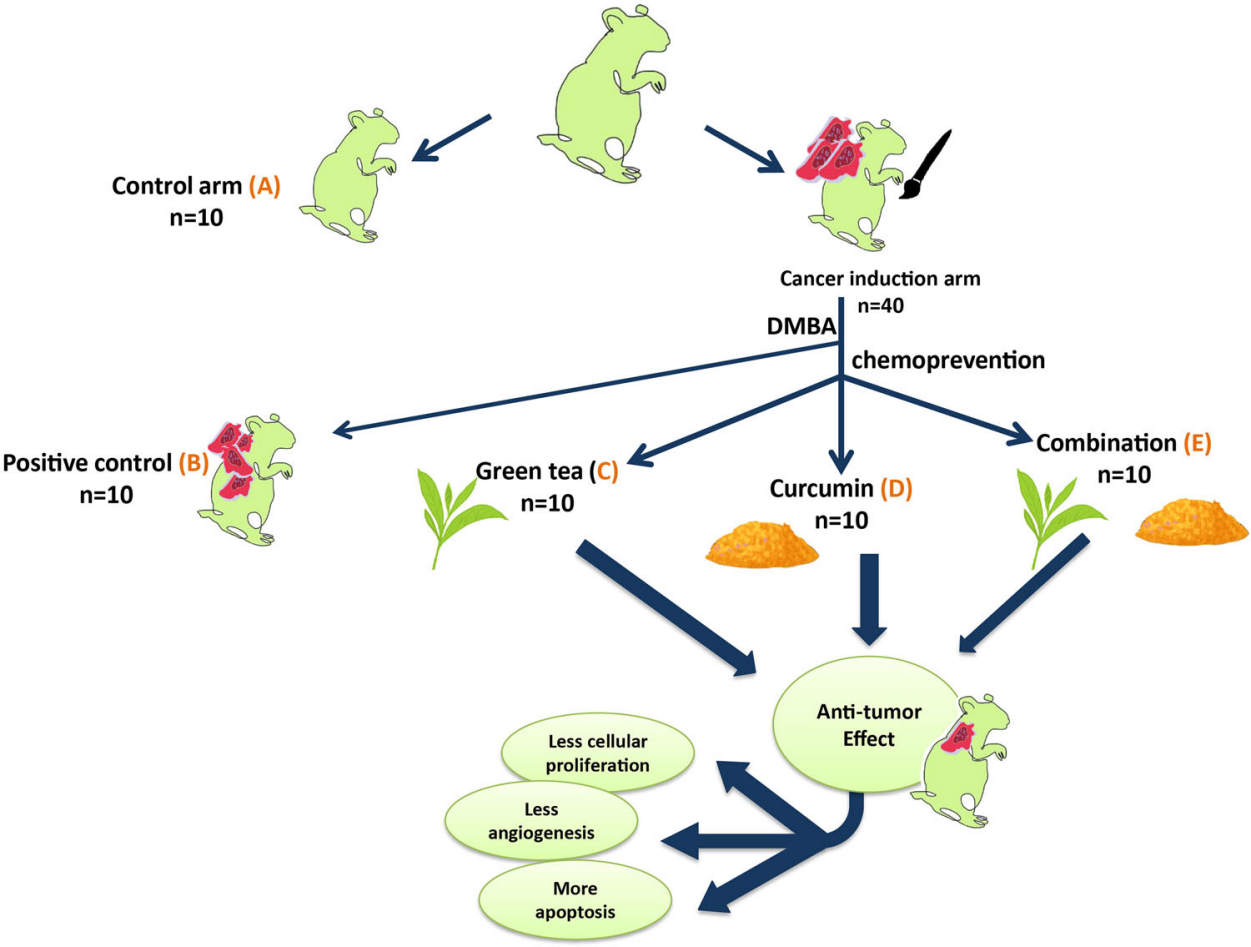
Graphical abstract. Graphical illustration of the study’s methodology and experimental steps.

Because we were aiming to assess the chemopreventive effect of green tea and curcumin on induced oral SCC so these agents were used at the same time as cancer induction. The carcinogenic agent (DMBA), green tea, and curcumin were used for 18 weeks. The animals’ left buccal pouches were painted with 0.5% DMBA dissolved in 100 ml liquid paraffin. A number 4 paintbrush was used for the application of the carcinogen. Following a standard carcinogenesis protocol, this procedure was repeated 3 times per week for 18 weeks. Each group (C, D, and E) received the proposed carcinogenic agent and the chemopreventive agents and was evaluated for 18 weeks. Green tea solids were added to the water and given to the hamsters as the sole source of drinking by gastric lavage. Curcumin in paraffin oil was applied topically on the left buccal pouch three times per week on alternative days with the carcinogen (Fig. 1).

### Clinical evaluation

After 18 weeks from starting treatment according to the previously published model protocol [24], the left buccal pouch of each animal was examined for clinicopathological changes. Animals were sacrificed by cervical decapitation under anesthetic conditions (Ketamin 30 mg/kg, i.p.). Finally, any animal disposals were burned.

Tumor tissue sections were extracted, and processed for the following:

### Histology and immunohistochemistry

After the hamsters were sacrificed, the biopsy was divided into 2 pieces. The first part of the biopsy taken from different animal groups was fixed in a 10% buffered formalin solution and embedded in paraffin wax. Sections 4 μm thick were mounted on glass slides stained with hematoxylin and eosin (H&E) from all the test groups and were evaluated under light microscopy by two independent pathologists and a consensus report was arrived to confirm the induced carcinoma or dysplasia.

For immunohistochemistry, the sections were mounted on positively charged glass slides, de-paraffinized, rehydrated, incubated with primary antibodies, and stained according to the manufacturer’s protocols. CD34 immunohistochemical marker was used to compare the microvessel density (MVD) among different study and control groups. The sections were examined by the image analyzer computer system using the software Leica Qwin 500. The device includes a light microscope cabled to a microcomputer that performs high-speed digital image processing. The number of microvessels in each group in ten fields at a magnification of ×400 in the area of the most intense vascularization (hot spot) was counted and the average count was recorded for each study group.

### Immunofluorescence analysis

Proliferating cell nuclear antigen (PCNA) immunofluorescence was used to compare cellular proliferation rates among control and study groups. The staining steps were done following the universal immunostain protocol. First, deparaffinization was done. The slides were placed in a rack, and the following washes were performed using a Coplins jar (Xylene (1 hour then 3 min), 1:1 Xylene:100% Ethanol (3 min), 100% Ethanol (3 min), 95% Ethanol (3 min), 70% Ethanol (3 min), and 50% Ethanol (3 min)). Once the slides were washed in this sequence, they were placed in running tap water to rinse off the ethanol. Second, permeabilization was performed in which the samples were incubated for 10 min in PBS containing either 0.1% Triton X. Then, the slides were washed for 3 × 5 min with Tris buffer solution (TBS) (PBS + 0.1% Tween 20) and were then blocked with 1% BSA in TBS for 1 h at room temperature. Slides were drained for a few seconds and the sections were wiped around using tissue paper.

The third step, blocking and immunostaining in which PCNA antibody (10 μg/mL.) was applied and diluted in TBS with 1% BSA and incubated overnight at 4 °C, then rinsed for 3 × 5 min TBS with gentle agitation. Fluorophore-conjugated secondary antibody (Alex Fluor 555) was applied to the slides diluted (1:400) in TBS with 1% BSA. Goat anti-mouse secondary antibody Alex Fluor 555(Invitrogen, Cat # A- 21422; Excitation/emission wave lengths 555/ 580 nm). The slides were then incubated for 1 hour at room temperature in the dark to avoid photobleaching, then rinsed for 3 × 5 min with TBS. At last, cells were incubated with 0.1 μg/mL Hoechst nuclear stain for 5 min. (Hoechst 33342 was used as a fluorescent nuclear stain (Thermo Fisher Scientific, Cat # 62249; Excitation /emission wavelengths 361/ 497 nm)) and rinsed 3 × 5 min with TBS, then mounted using a compatible mounting medium and then covered with a coverslip.

The examination was done by confocal laser scanning (CLS); Leica TSC SPE II/ DMi 8, a unit of Advanced Microscopy, at CERMA. The Imaging was done by applying both the fluorescent and differential interference contrast (DIC) modes either individually for each fluorochrome or in a merged pattern. The DIC mode is an optical system for retrieval of information obtained by phase-contrast imaging while providing narrow optical sections by CLS; thus, rejecting out-of-focus information and hence maintaining high phase contrast resolution for transparent objects.

### FACS assay for apoptosis analysis

The second part of tumor cells isolated from primary tumors of the induced buccal SCC was preserved in Roswell Park Memorial Institute (RPMI) 1640 tissue culture media. The fresh tissue specimens were homogenized by thoroughly mincing with sharp surgical blades in cold RPMI 1640 medium and trypsin/ EDTA on sterile disposable Petri dishes. The extracted cells were separated from the remaining tissue by filtration through a 100 µm meshed nylon cell strainer in a sterile Falcon tube.

The cell extracted was centrifuged (2000 rpm, 20 min), and incubated with 1 ml trypsin enzyme for 20 min. Re-centrifugation (2000 rpm, 5 min) was done to remove the trypsin enzyme, followed by 2 washes using phosphate buffer saline and bovine serum albumin (PBS, BSA) with centrifugation for 5 min after each wash. Fixation of isolated cells was performed using 70% ice-cold ethanol and vortexing to prevent cell clumping, followed by cry freezing at −20 °C. On thawing, the cells were left to reach room temperature, and centrifuged for 5 min to remove the excess alcohol, followed by 2 washes using PBS and BSA with centrifugation for 5 min after each wash. Further purification by re-filtration was done using 100 µm meshed nylon cell strainers mesh to obtain a single cell suspension and to remove any tissue clumps.

Incubation with Annexin V conjugated to FITC fluorochromes was used for the assessment of early-phase apoptosis. Annexin V serves as a sensitive probe for quantitative flow cytometric analysis of the cells that are undergoing apoptosis.

### Acquisition and data analysis

Acquisition and data analysis of the stained cell suspensions were performed at the Center of Excellence for Research in Regenerative Medicine Applications, CERRMA, Faculty of Medicine, Alexandria University using a fluorescent-activated cell sorter (FACS) Calibur flow cytometer (Becton Dickinson, San Diego, Calif, USA) and Cell Quest software (Becton Dickinson) respectively. The cells were analyzed within 4 h after the initial incubation period to avoid any adverse effects on the viability of the cells left in the presence of the PI for a long time period. The samples were run in duplicate with about 10,000 events counted per tube.

### Statistical analysis

All data were collected; tabulated and statistically analyzed using the SPSS system (Statistical Package for Scientific Studies). ANOVA test was used to analyze the data between the study groups.

A (P) value less than 0.05 was considered significant. The values were given as a mean value ± SD (standard deviation).

## Results

In the current study, a total of 50 male Syrian gold hamsters were included. After 18 weeks, the left buccal pouches were examined to detect any pathological changes. Clinical examination, histological findings, immunohistochemical staining, immunofluorescence, and flow cytometry for apoptosis detection were evaluated.

### Clinical and histological evaluation in hamster buccal pouch carcinoma (HBPC) model

Clinical assessment of the animals indicated the development of different lesions including leukoplakia, erythroplakia, and exophytic carcinoma during the time course of the study. Histological assessment of the tissues from the HBPC model showed progression of the disease between the 0th and 18th week with 100% of the samples being diagnosed.

**Fig. 2:**
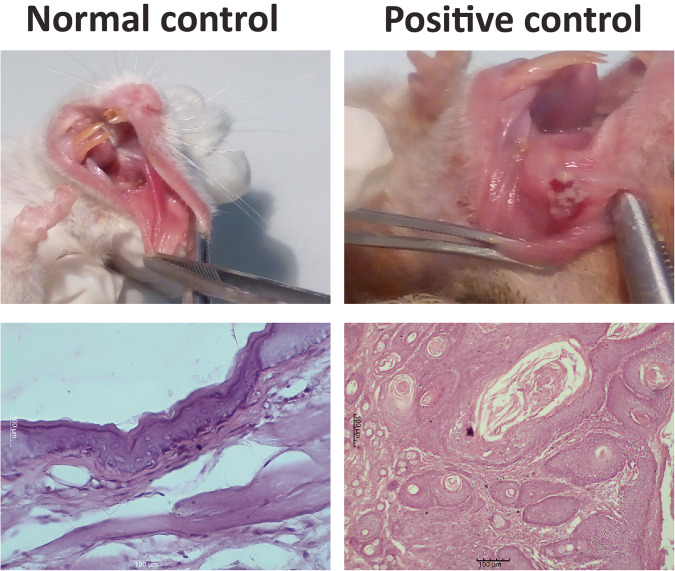
Normal HBP mucosa appearing pale pink showing no pathological changes. A photomicrograph showing flat keratinized squamous epithelium without rete ridges (H&E X400) (Group A). In the positive control, the HBP showing red and white exophytic mass. A photomicrograph showing well differentiated SCC (H&E X100) (Group B).

DMBA group (positive control) showed well-developed oral exophytic lesions in all HBP (100%). Seven hamsters exhibited red lesions and three hamsters showed evident red and white lesions. Different tissue biopsies were evaluated. Four biopsies revealed well-differentiated oral SCC where malignant epithelial cells formed keratin and epithelial pearls. The other six biopsies showed moderately differentiated oral SCC where malignant epithelial cells formed epithelial nests and few keratin pearls. Malignant criteria such as pleomorphism, hyperchromatism, and abnormal mitotic figures were detected. Comparison with the normal control group showed that the lining epithelium of HBP mucosa is flat keratinized stratified squamous epithelium without rete ridges. The subepithelial connective tissue consisted of dense fibrous tissue without any tumor tissue (Fig. 2).

### Clinical and histological responses on treatment with green tea and curcumin

After the 18th week, the animals (*N* = 10 in each group) were grouped into different arms, treated with the chemopreventive agents (individual/combination), and assessed for their clinical response. The green tea group revealed white patches in seven hamsters (70%), while two hamsters (20%) revealed small exophytic lesions. In only one hamster (10%), no pathological changes were seen. The curcumin group showed red patches in five hamsters (50%) and three hamsters (30%) revealed small exophytic lesions. No pathological changes were seen in the remaining two hamsters (20%). The combination group showed white patches in seven hamsters (70%), while three hamsters (30%) revealed only tiny red exophytic lesions.

**Fig. 3:**
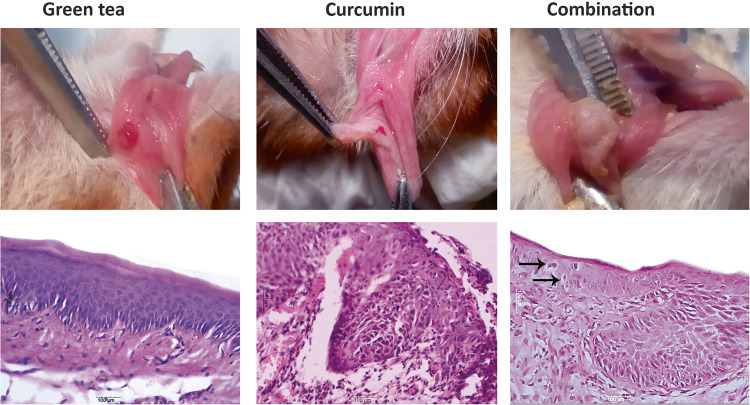
In green tea group, The HBP showing a small red exophytic mass. A photomicrograph showing basilar hyperplasia of the surface epithelium (H&E X400) (Group C). Curcumin group, a red patch was seen in the HBP. A photomicrograph showing severe dysplastic epithelium with loss of cohesiveness of the malignant epithelial cells (H&E X400) (Group D). Combination group, the HBP showing a whitish leukoplakic mass. A photomicrograph showing moderate epithelial dysplasia and several apoptotic bodies (arrows) (H&E X400) (Group E).

Histologically, in the green tea group, dysplastic changes in the epithelial layer of seven biopsies were detected while two hamsters showed well differentiated SCC. Only one showed no pathological changes in the lining epithelium. The Curcumin group revealed moderate to severe epithelial dysplasia in five biopsies while three biopsies showed evident invasive carcinoma. No pathological changes in the lining epithelium of the remaining two biopsies were seen. Compared to the combination group, moderate to severe epithelial dysplasia with areas of top to bottom changes or carcinoma in situ were seen in seven biopsies while three biopsies showed well-differentiated SCC (Fig. 3).

### Combination treatment decreased the expression of CD34

**Fig. 4:**
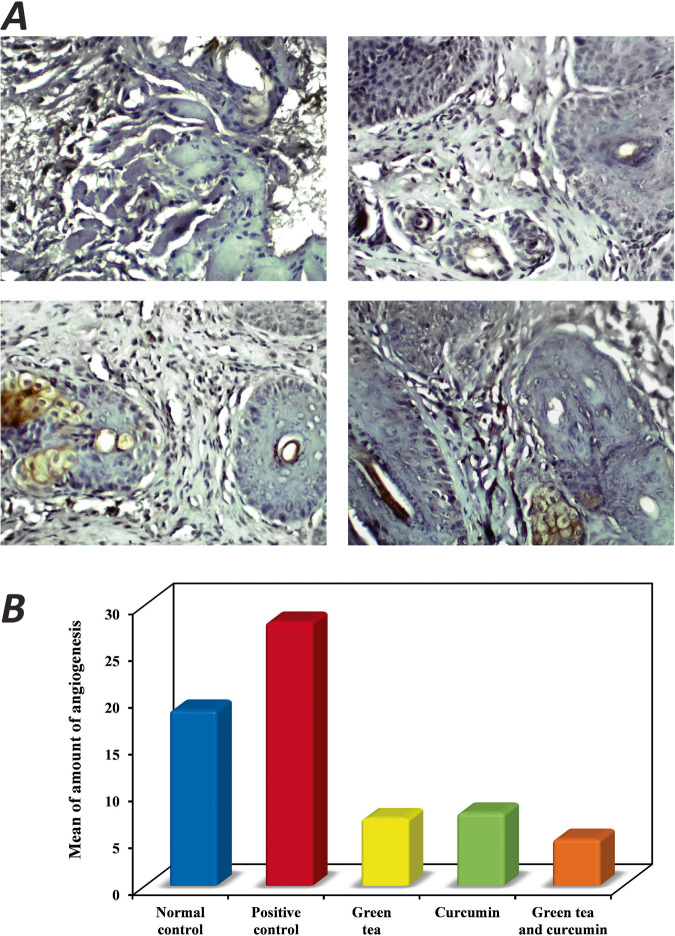
Immunohistochemical expression of CD34 in different study groups. **A** Immunohistochemical image demonstrated the immunopositivity among different study groups. The combination group showed the lowest immunopositivity compared with green tea and curcumin individually (CD34 X400). **B** Graph represents comparison between the different studied groups according to amount of angiogenesis.

Assessment of CD34 immunohistochemically across the different treatment arms (negative control: 10, positive control: 10, green tea: 10, curcumin: 10, and combination: 10) showed that the expression of CD34 was lowered in the green tea arm as compared to the positive control arm (MVD: 7.20 ± 0.92 vs 28.20 ± 6.41). Also, the curcumin arm showed decreased immunopositivity (7.80 ± 2.90) and the combination arm (5.0 ± 0.94) revealed the lowest immunoreaction compared with green tea and curcumin individually though the difference was not statistically significant. Although the mean was higher in the positive control arm as compared to the treated arms (Fig. 4).

### Green tea and curcumin downregulated the cellular proliferation

Immunofluorescence analysis was used to assess cellular proliferation among the control and treatment groups. First, the DIC (Differential Interference Contrast) mode demonstrated the outline of the cells in the control and treatment groups. Second, the fluorescent images labeled for the detection of PCNA in cancer cells indicated cell proliferation.

The DIC mode of the normal control group(group A) revealed the surface epithelium and the underlying highly vascular connective tissue with no evidence of tumor cells while positive control (group B) revealed nests of SCC invading the underlying connective tissue. The cells were pleomorphic in shape with extending irregular cytoplasmic processes. The DIC mode for green tea, curcumin, and combination (group C, D, and E respectively) showed nests of SCC invading the underlying connective tissue with variable sizes.

Furthermore, the fluorescent image of group A showed the DNA of the cells as blue fluorescence with no red fluorescence indicating no proliferation in the normal control group while group B showed increased expression of the red fluorescence in the nucleus and cytoplasm of the squamous cell carcinoma cells indicating highly proliferating cells.

**Fig. 5:**
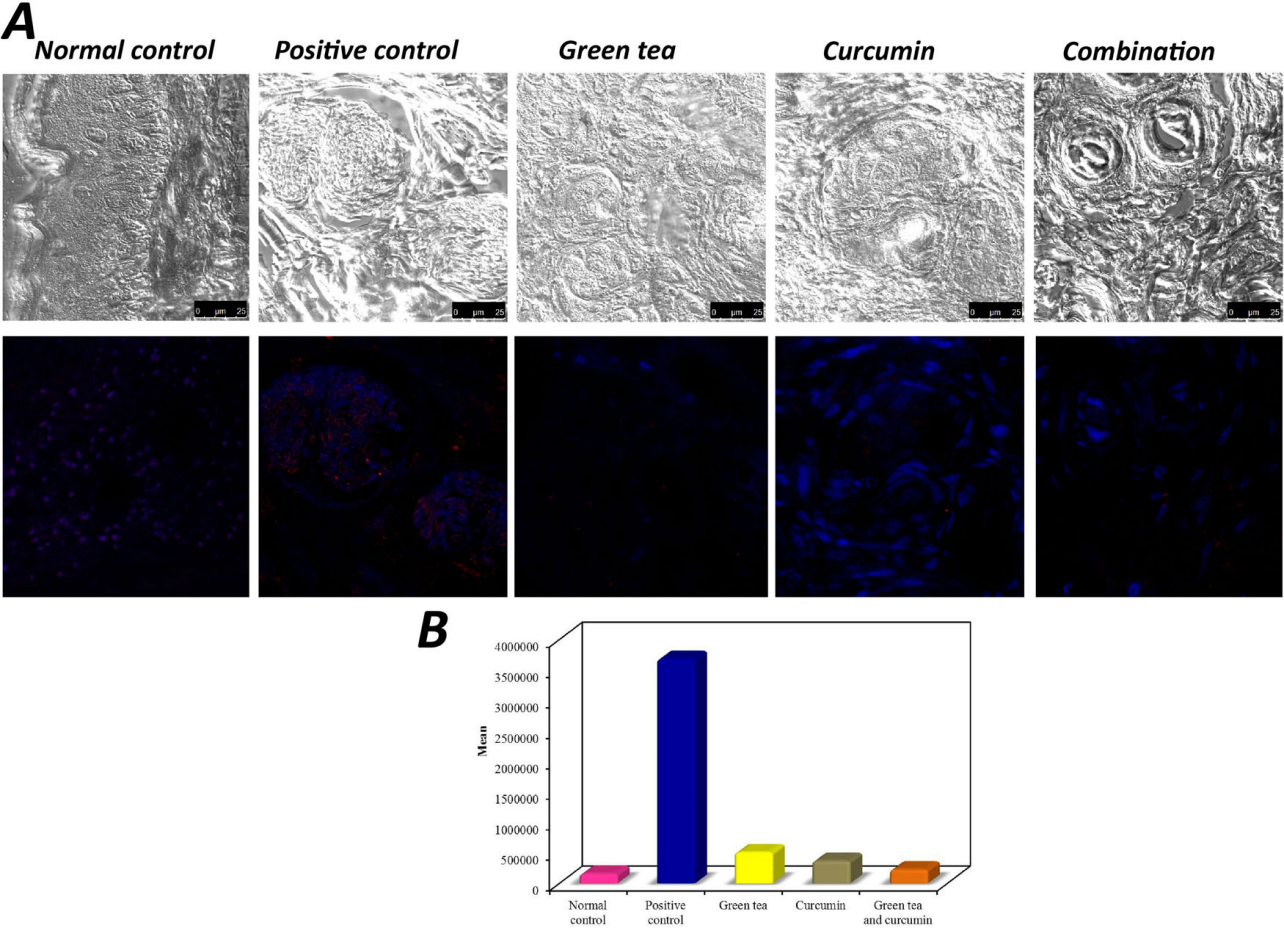
Immunoflouresence analysis of PCNA in study groups. **A** Confocal Laser Scanning Photomicrograph of normal control group showing the surface epithelium and the underlying connective tissue by The Phase Contrast Mode (DIC), the fluorescent image showing the DNA of the cells by blue fluorescence. Confocal Laser Scanning Photomicrograph of positive control group showing nests of SCC invading the underlying connective tissue by DIC, the fluorescent image showing red fluorescence in the nucleus of the SCC cells. Confocal Laser Scanning Photomicrograph of green tea group showing small nests of SCC with blood vessel invasion by DIC, the fluorescent image showing red fluorescence in the nuclei of the SCC cells. Confocal Laser Scanning Photomicrograph of curcumin group showing large nest of SCC with irregular shaped large and small cells by DIC, the fluorescent image showing red fluorescence in the nuclei of the SCC cells. Confocal Laser Scanning Photomicrograph of the combination group showing nests of SCC with depressed area of the center revealed keratin by DIC, the fluorescent image showing red fluorescence in the nuclei of the SCC. **B** Graph represents comparison between the different studied groups according to cell proliferation using PCNA.

The fluorescent image of groups C, D, and E showed decreased expression of the red fluorescence in the nuclei of the squamous cell carcinoma cells indicating low cellular proliferation (Fig. 5).

### Induction of apoptosis by combination treatment

The extracted cells from different study groups were separated from the remaining tissue. Incubation with Annexin V serves as a sensitive probe for quantitative flow cytometric analysis of the cells that are undergoing apoptosis. The flow cytometry profiles were displayed as a four-quadrant scatterplot. The profile graph presented the Annexin V FITC parameter along the X-axis and the PI (Propidium iodide) parameter along the Y-axis. The four quadrants of the scatterplot are presented, in a clockwise direction starting from the upper left quadrant: the population of necrosed cells, cells in late apoptosis, cells in early apoptosis, and the living cells, respectively. Of these four kinds of cell modes, early and late apoptosis were calculated among the cell population.

**Fig. 6:**
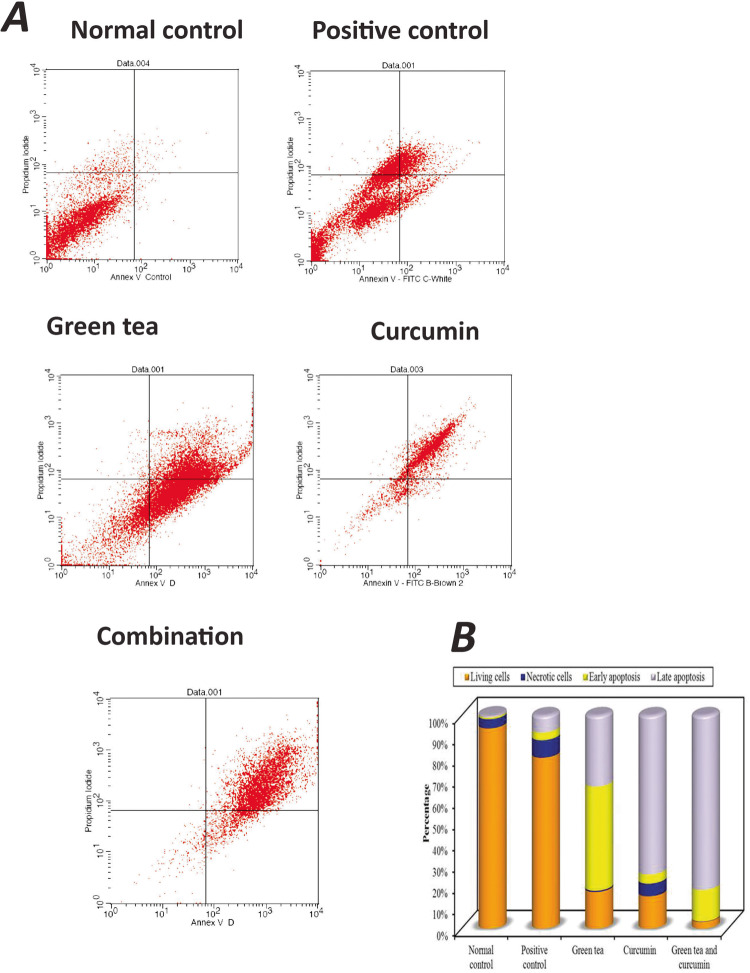
Flow Cytometryt analysis. **A** Flow Cytometry Scatter Plots for Annexin V-FITC and PI Staining to Evaluate Apoptosis in the Different Groups. Normal control, Positive control, Cells treated with green tea, Cells treated with curcumin, Cells treated with both green tea and curcumin. **B** Graph showing summary of the Annexin V-FITC Apoptosis Assay Results.

In the untreated cells (normal control), 1.72% of the cells underwent apoptosis (0.81% early apoptosis, 0.91% late apoptosis) while the cells of the positive control group resulted in 11.57% apoptosis (3.59% early apoptosis, 7.98% late apoptosis) and only 8.16% necrosis. In the study groups, treatment of the cells with green tea resulted in 82.22% apoptosis (48.86% early apoptosis, 33.36% late apoptosis) and 0.55% necrosis while treatment of cells with curcumin resulted in 78.91% apoptosis (4.25%early apoptosis, 74.66% late apoptosis) and 5.87% necrosis. Cells treated with both green tea and curcumin resulted in 96.63% apoptosis (14.70% early apoptosis, 81.93% late apoptosis) and 0.20% necrosis (Fig. 6).

## Discussion

Oral squamous cell carcinoma is a major burden of morbidity and mortality with a poor 5-year survival rate [28]. As a result of the limitations of conventional therapeutic approaches in some difficult cases, chemoprevention has evolved as a promising strategy to inhibit, suppress or regulate the incidence of carcinogenesis by using specific natural and synthetic compounds [29]. Green tea and curcumin, which contain significant quantities of polyphenols, are considered promising natural chemopreventive agents against oral carcinogenesis [1]. Induction of apoptosis, together with a decrease in cell proliferation and angiogenesis are modulated by chemopreventive drugs [24].

It has been demonstrated in the present study that green tea and curcumin have chemopreventive effects (each individually and in combination) on induced hamster buccal pouch carcinoma. We hypothesized that using a combination of the two drugs may improve chemoprevention efficacy on apoptotic, proliferative, and angiogenic pathways. Because of the similarities in the mechanisms involved, such as the formation of DNA adducts and subsequent genetic changes, the HBPC oral cancer model is considered a proxy for tobacco-induced carcinogenesis. Furthermore, all of the animals in this model develop oral cancer through premalignant lesions, making it a useful model system for testing the chemoprevention efficacy. In this study, this model was used for evaluating the clinical efficacy and molecular basis of combination treatment with green tea and curcumin. This model is crucial for understanding the underlying molecular basis of oral cancer and developing chemopreventive treatments. A previous study in the HBPC model has reported the efficacy of green tea and curcumin individually and in combination. It was suggesting a possible cross-talk and synergic effect of two drugs on the targeted pathways [24]. In this study, combination treatment with green tea and curcumin led to improved efficacy of chemoprevention at a clinical level in the hamster buccal pouch-induced carcinoma. The drug combination could, however, elicit only a moderate histological response. Whether the change in dosage, duration, or mode of drug administration can result in improving the same, needs to be investigated.

It was found that green tea promotes apoptosis (82.22%) and causes cell cycle deregulation which is in agreement with Thambi Dorai [30]. The anti-cancer effects of catechins may be related to the production of oxidative stress and the stimulation of apoptosis in tumor cells caused by these pro-oxidant actions. These pro-oxidant actions may also activate endogenous antioxidant defense mechanisms in healthy tissues, which provide defense against carcinogenic damage. The mechanism underlying the anticarcinogenic effects of catechins involves inhibiting the proliferation and growth of cancer cells, scavenging free radicals, suppressing metastasis of cancer cells, improving immunity, and regulating signaling pathways [31]. Other studies showed that green tea may be more effective post-initiation, suppressing early lesions by inhibiting cell growth in the rat colon. In the intestinal mucosa of rats, it dramatically reduced cyclin D1 levels [32, 33]. In 2012, Henning et al. demonstrated that using aqueous-brewed green tea extract decreased VEGF (Vascular Endothelial Growth Factor) in mice with prostate cancer cell subcutaneous xenografts. After 11 weeks, the VEGF protein expression was reduced by 35% [34]. The application of curcumin in our HBPC model also suppressed cell cycle arrest and cell proliferation and induced apoptosis (78.91%). Curcumin induces apoptosis through cytochrome c release, activation of caspase 3, caspase 8, and DNA fragmentation. Curcumin was able to modulate several cellular pathways affecting proliferation and/or angiogenesis, invasion, migration, metastasis, and apoptotic processes. Change in the expression of tumor suppressor, pro-apoptotic, and anti-apoptotic genes by curcumin represents one of the key molecular mechanisms underlying its anti-cancer action [35]. Brandon also demonstrated that in the buccal pouch of hamsters exposed to the carcinogen Benzo[a]pyrene, 0.6% curcumin inhibited cellular proliferation, p53 accumulation, and induction of apoptosis [36]. Previous studies suggested that the ratio of Bax/Bcl2 is a crucial component in determining whether a cell will die or survive [37]. Moreover, in vivo study demonstrated that PCNA is over-expressed during DMBA-induced HBP carcinogenesis and that curcumin dramatically reduced DMBA-induced PCNA levels over the course of the study [38]. Curcumin also prevented VEGF and MMP-2 production in laryngeal squamous cell carcinoma cells by reducing JAK2 and STAT3 phosphorylation and inhibiting angiogenesis [39].

Curcumin’s supraadditive effect when combined with currently prescribed chemotherapeutic agents, radiotherapy, novel therapeutic options, or even other plant-derived substances has prompted a number of studies with various cancer types, all of which found an additive effect when compared to treatment alone. Curcumin was found to have similar effects when combined with EGCG, resveratrol, and photodynamic treatment in head and neck SCC cell lines and animal models [40]. Our research showed synergistic apoptosis induced by the combination of green tea and curcumin. Either green tea or curcumin is a strong inhibitor of cell cycle kinases and cell cycle related proteins. Co-treatment of the two natural compounds may simultaneously affect multiple cell cycle-related target pathways. To determine whether co-treatment of green tea and curcumin induced cancer cell cycle arrest, flow cytometry analysis was performed to measure the distribution of cell cycles. Green tea mainly arrested the S phase however curcumin arrested the G2/M phase. Arrests in both the S and G2/M phases of the cell cycle were observed by the co-treatment of both green tea and curcumin. Therefore, the combined beneficial effect of the two agents was mediated by the induction of their different actions on cell cycle arrest [41]. Recent evidence explored the mechanism of synergistic apoptosis induced by the combination of green tea and resveratrol. Green tea, resveratrol, and their combination decreased the expression of Mcl 1 and the survival of cells, according to their findings [22]. Anti-apoptotic Bcl-2 proteins such as Bcl-2, Bcl-xL, and Mcl-1 act as mitochondrial gatekeepers and ensure the integrity of the mitochondrial membrane. By lowering mitochondrial membrane potential and permitting the release of cytochrome c in the cytoplasm to trigger the apoptotic cascade, inhibition of the production of these anti-apoptotic Bcl 2 proteins favors apoptosis. In agreement with the previous results which concluded that green tea or curcumin were both effective inhibitors of cell cycle-related proteins and kinases. The combination of these two chemicals may be able to target numerous cell cycle pathways at the same time. They discovered that green tea coupled with curcumin reduced cyclin B1 and cyclin D1, resulting in a considerable G1 and S/G2 phase arrest [42]. Furthermore, similar results were reported in prostate cancer whereas green tea or curcumin are both powerful inhibitors of cell cycle kinases and associated proteins. However, the distinctions in how both agents work at different stages of the cell cycle are unknown [41]. Li et al., on the other hand, discovered that the inhibitory effect of green tea and curcumin on angiogenesis was not evident in all of their study’s lesions [24].

Because of the increased efficacy at the early stages of tumor development, intervention at the onset of carcinogenic transformation is likely to be more advantageous than intervention at later stages, when the balance is skewed in favor of the aggressive tumorigenic machinery. So better results were achieved when administration of the chemopreventive agents at the initiation/post-initiation stage of carcinogenesis. Although curcumin has low toxicity in humans and animals, it has a variety of limitations when it comes to therapeutic usage, particularly in terms of solubility and bioavailability [43]. In vivo, an investigation carried out in this study points out green tea and curcumin being an efficient combination for chemoprevention as demonstrated by the clinical response, histological, immunohistochemical, immunofluorescence, and flow cytometry assays. Further targeting molecular pathways is essential to establish the chemopreventive potential of this approach.

## Conclusions

Green tea used along with curcumin as a chemopreventive agent shows synergistic growth inhibitory effects and superior anti-tumor effects on induced carcinogenesis from the aspect of their apoptotic, anti-proliferative, and anti-angiogenic action on tumor cells by suppressing a variety of cellular signaling pathways, enzymatic activity, and protein kinases using flow cytometry, immunofluorescence, and immunohistochemical assays. They are considered promising adjuvant chemopreventive agents to prevent the progression of OSCC.
